# Fluoroquinolones Suppress TGF-β and PMA-Induced MMP-9 Production in Cancer Cells: Implications in Repurposing Quinolone Antibiotics for Cancer Treatment

**DOI:** 10.3390/ijms222111602

**Published:** 2021-10-27

**Authors:** Cheng-Yi Huang, Jenq-Lin Yang, Jih-Jung Chen, Shun-Ban Tai, Yu-Hsuan Yeh, Pei-Feng Liu, Ming-Wei Lin, Chih-Ling Chung, Chun-Lin Chen

**Affiliations:** 1 Department of Biological Sciences, National Sun Yat-Sen University, Kaohsiung 80424, Taiwan; zyhuang600@gmail.com (C.-Y.H.); taisunban@gmail.com (S.-B.T.); yeh0935135650@gmail.com (Y.-H.Y.); chungling810@gmail.com (C.-L.C.); 2 Department of Pathology, Kaohsiung Armed Forces General Hospital, Kaohsiung 80284, Taiwan; 3 Institute for Translational Research in Biomedicine, Kaohsiung Chang Gung Memorial Hospital, Kaohsiung 83301, Taiwan; jyang@cgmh.org.tw; 4 Department of Pharmacy, School of Pharmaceutical Sciences, National Yang Ming Chiao Tung University, Linong Street, Taipei 11221, Taiwan; jjungchen@nycu.edu.tw; 5 Department of Medical Research, China Medical University Hospital, China Medical University, Taichung 40402, Taiwan; 6 Division of Rheumatology, Immunology and Allergy, Department of Internal Medicine, Zuoying Branch of Kaohsiung Armed Forces General Hospital, Kaohsiung 81342, Taiwan; 7 Department of Biomedical Science and Environmental Biology, Kaohsiung Medical University, Kaohsiung 80756, Taiwan; pfliu@kmu.edu.tw; 8 Department of Chinese Medicine, E-Da Hospital, Kaohsiung 82445, Taiwan; ed110208@edah.org.tw; 9 Department of Biotechnology, Kaohsiung Medical University, Kaohsiung 80708, Taiwan; 10 Graduate Institute of Natural Products, College of Pharmacy, Kaohsiung Medical University, Kaohsiung 80756, Taiwan

**Keywords:** fluoroquinolones, matrix metalloproteinase-9, TGF-β, EMT

## Abstract

Background: Fluoroquinolones (FQs) are potent antimicrobials with multiple effects on host cells and tissues. Although FQs can attenuate cancer invasion and metastasis, the underlying molecular mechanisms remain unclear. Matrix metalloproteinase-9 (MMP-9) has functional roles in tumor angiogenesis, invasion, and metastasis, and is associated with cancer progression and poor prognosis, suggesting that inhibitors of MMP-9 activity and transcription are prime candidates for cancer therapy. Despite numerous preclinical data supporting the use of MMP-9 inhibitors as anticancer drugs, the few available examples are not therapeutically useful due to low specificity and off-target effects. We examined the effects of FQs on MMP-9 production in cancer cells following transforming growth factor beta (TGF-β) and phorbol 12-myristate 13-acetate (PMA) stimulation. Experimental approaches: Using confluent cultures of HepG2 and A549 cells, the effects of FQs (ciprofloxacin, levofloxacin, clinafloxacin, gatifloxacin, and enrofloxacin) on TGF-β and PMA-induced MMP-9 mRNA expression and production were studied in RNA extracts and culture supernatants, respectively. FQs specifically abrogated TGF-β and PMA-induced MMP-9 levels and activity in a concentration and time-dependent manner, without affecting other MMPs or proteins involved in epithelial-mesenchymal transition. Additionally, FQs inhibited TGF-β and PMA-induced cell migration via p38 and cyclic AMP signaling pathways. Conclusions and implications: Overall, we demonstrated that FQs inhibit cancer cell migration and invasion by downregulating MMP-9 expression and revealed the cellular mechanisms underlying their potential value in cancer treatment.

## 1. Introduction

Tumor metastasis is the primary cause of cancer-related morbidity and mortality [1]. New anticancer agents that prevent malignant transformation, invasion, and metastasis of cancer cells have recently attracted attention [2]. Tumor invasion and metastasis are multi-step processes that include the destruction of the extracellular matrix (ECM), detachment of cells from the original cancer site, attachment to ECM binding sites, and migration to target tissues [3]. Cancer cells invade tissues at distant metastatic sites by using matrix metalloproteinases (MMPs) to degrade a variety of ECM proteins [3]. Among these MMPs, MMP-2 and MMP-9 are zinc-containing gelatinases that have been shown to induce tumor progression by promoting cell invasion and metastasis [4]. Thus, the inhibition of MMP-2 and MMP-9 activities can be employed as an important anti-metastasis strategy to prevent cancer cell dissemination. Although these two MMPs have similar properties, their gene expression is specifically regulated by different regulatory elements in their respective promoter regions [5].

In contrast to MMP-2, which is constantly expressed in non-malignant cells and tumor cells, MMP-9 is strongly associated with the malignant progression of various cancers and is highly inducible in response to a variety of stimuli, including transforming growth factor beta (TGF-β), epidermal growth factor (EGF), phorbol 12-myristate 13-acetate (PMA), ultraviolet radiation, and oncogenes [6,7,8]. Among these inducers, PMA and TGF-β can increase MMP-9 expression and secretion during cancer cell invasion by activating nuclear factor kappa B (NF-κB) and activator protein 1 (AP-1), as their promoter region contains binding sites for these transcription factors [9]. Although MMP-9 is strongly regarded as a promising target for cancer treatment, MMP-9 inhibitors have thus far failed in clinical trials due to serious side effects and no therapeutic benefit [10,11,12]. Because of the structural similarity among MMPs, many such inhibitors have limited ability to discriminate between various MMPs, and developing improved selectivity has proven challenging [12,13]. These conundrums prompted us to use the Johns Hopkins Drug Library, which contains approximately 3000 drugs, for high-throughput screening. Among the hits, fluoroquinolones (FQs) were identified as potent inhibitors of MMP-9.

FQs are broad-spectrum antibiotics with few side effects; they exert their antibacterial activity by stabilizing the ternary complex of bacterial DNA gyrase, which is responsible for extensive DNA fragmentation and bacterial death [14]. FQs also display non-canonical mechanisms of action including, but not limited to, immunomodulatory and antitumor properties. Sparfloxacin was shown to suppress MMP-9 production by blocking HERG channel [15]. Additionally, by affecting p21/p27/p53 activities, gatifloxacin was also found to synergize the effect of gemcitabine, cisplatin as well as the broad-spectrum anticancer drugs against pancreatic cancer [16]. Kloskowski et al. demonstrated that anticancer effect of ciprofloxacin on five cell lines including human non-small cell lung cancer line A549, human hepatocellular carcinoma line HepG2, human and mouse melanoma lines (A375) and rat glioblastoma line C6 [17]. Moreover, several derivatives of ciprofloxacin have been shown to exhibit more potent antitumor activity than the parent compound, including analogs whose inhibitory potency (IC_50_) values are lower than 10 μM in various cancer cell lines [14,18]. These non-antimicrobial effects of FQs are unique among antibiotics and suggest a broad spectrum of future clinical applications.

Although the mechanisms of antibacterial, antitumor, and immunomodulatory activity of FQs have been broadly investigated in vitro and in vivo [19,20], the potential mechanisms of FQs against tumor invasion and metastatic activity have not been elucidated in a comprehensive and satisfying manner. FQs possess immunomodulatory properties due to their effects on phosphodiesterase and the generation of intracellular cyclic AMP (cAMP) [19,20,21,22]. It has been proposed that cAMP, a second messenger, can serve as an anti-inflammatory agent by activating downstream signaling pathways, such as protein kinase A (PKA) and the exchange protein directly activated by cAMP (Epac), and inhibiting the secretion of cytokines [21]. Moreover, recent data provide evidence that the modulation of intracellular cAMP levels can also regulate some cellular properties involved in cell motility [23,24]. From the perspective of signal transmission, it has been demonstrated that the active crosstalk between cAMP and the p38 signaling pathway allows cAMP to modulate p38 depending on the cell type and cellular environment [25,26,27,28,29].

TGF-β exerts pleiotropic effects on cancer progression by regulating cell growth, differentiation, migration, ECM remodeling, and by modulating the immune response. Among the growth factors involved in tumor development, TGF-β is of particular interest, given its prominent role in all phases. TGF-β-induced tumor invasion and metastasis are closely related to the proteolytic activity of MMP-9. Active signaling occurs via a heterodimeric serine-threonine kinase receptor composed of TGF-β receptor I (TβR-I) and TGF-β receptor II (TβR-II). In canonical TGF-β signaling, TGF-β homodimers bind to TβRII, leading to heterodimerization with TβRI; TβRII phosphorylates TβRI, which in turn propagates the signal to the transcription factors Smad2 and Smad3 [30]. Binding of phosphorylated Smad2/3 to cytoplasmic Smad4 allows translocation into the nucleus and regulation of transcription. Although TGF-β signaling occurs mainly via the Smad pathway, it can also activate other pathways that are collectively referred to as ‘non-canonical’ TGF-β signaling pathways that complement the Smad pathway, such as mitogen activated protein (MAP) kinase, p38 MAP kinase, Rho GTPases, and PI3 kinases [30]. PMA is a high-affinity ligand and activator of conventional protein kinase C (PKC) and its isoforms [31]. It strongly stimulates MMP-9 expression via PKC in several subsequent mediators, including extracellular signal-regulated kinase (ERK) 1/2 and p38 MAP kinase [32,33,34].

In this study, we demonstrated the anti-invasive potential of FQs using human-derived lung adenocarcinoma (A549) and hepatocellular carcinoma cell lines (HepG2) with invasive phenotypes. To mimic the physiological effects of MMP-9 and explore the potential signaling pathways involved in the inhibitory effect of FQ on MMP-9 production in the cell systems, we used TGF-β and PMA to induce MMP-9 secretion. Our results show that FQs effectively inhibited the in vitro invasiveness of A549 cells via downregulation of MMP-9 expression, mediated by the effect of FQ on p38 and intracellular cAMP.

## 2. Results

### 2.1. FQs Inhibited Cancer Cell Migration and Invasion

**Figure 1:**
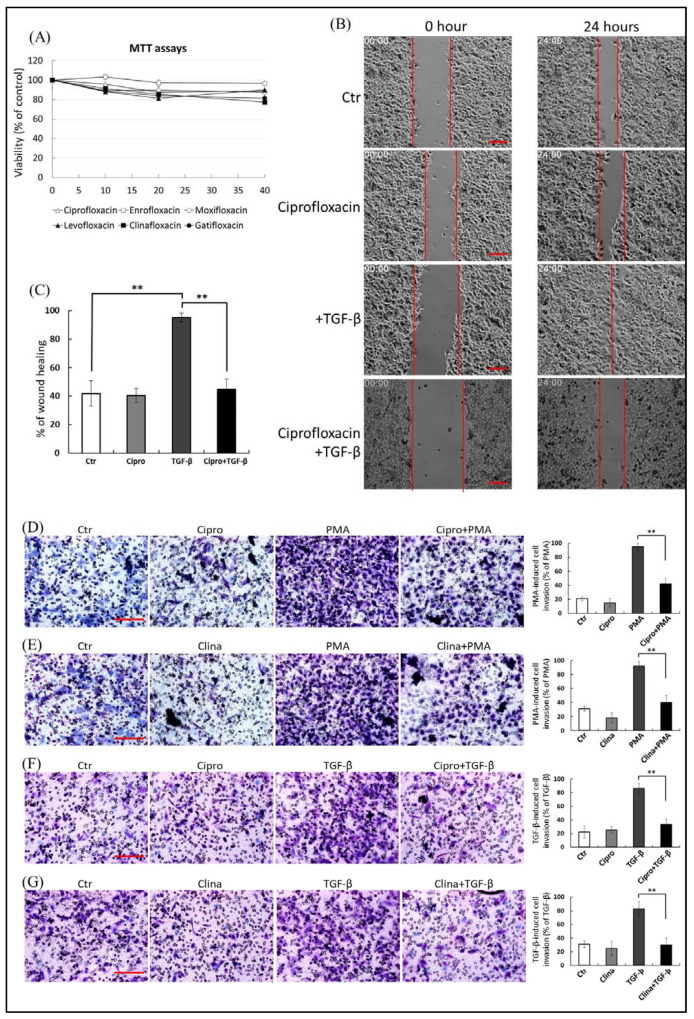
(**A**) A549 cells (1 × 10^4^/well in a 96-well plate) were treated with 1–40 μM of fluoroquinolones (FQs; ciprofloxacin, enrofloxacin, moxifloxacin, levofloxacin, clinafloxacin, and gatifloxacin) for 48 h. Cell viability of cells was determined by the 3-(4,5-dimethylthiazol-2-yl)-2,5-diphenyltetrazolium bromide assay. Each FQ treatment group was normalized to each un-treated control. The data (mean ± standard deviation, SD) are representative of three independent experiments; error bars indicate SD. (**B**,**C**) Ciprofloxacin inhibits transforming growth factor beta (TGF-β)-stimulated cell migration in HepG2 cells. A fully automated time-lapse microscope was used to perform wound healing measurements under a 10× phase objective lens, capturing images of all four experimental conditions simultaneously every 20 min. Images were captured from 0–24 h in serum-free medium in the presence of ciprofloxacin (20 μM) or an equal volume of dimethyl sulfoxide (DMSO). Representative photomicrographs of the three experiments are shown in panel (**B**, Bar = 400 μ). Panel (**C**) illustrates the quantitative analysis of cell coverage (mean ± SD) based on three independent experiments. The percentage wound closure was plotted over 24 h under each condition; the wound area was normalized to the initial value at 0 h. (**D**) Suppressive effect of ciprofloxacin on phorbol 12-myristate 13-acetate (PMA)-induced A549 cell invasion. (**E**) Suppressive effect of clinafloxacin on PMA-induced A549 cell invasion. (**F**) Inhibition of ciprofloxacin on TGF-β-stimulated A549 cell invasion. (**G**) Inhibition of clinafloxacin on TGF-β-stimulated A549 cell invasion, Bar = 400 μ. A549 cells in serum-free medium containing FQs were seeded onto the upper chambers of Matrigel-coated filter inserts for 1 h, followed by incubation with or without 10 nM PMA (**D**,**E**) or 200 pM TGF-β (**F**,**G**) for another 24 h. Cells that had been invaded were stained and quantified as the percentage of cell invasion relative to that of invasion of PMA or TGF-β-stimulated experiments, the results of which are shown in the right-hand graphs; the data are presented as means ± SD (error bars) of three independent experiments. ** p ≤ 0.01 vs. PMA or TGF-β-treated group. Representative microscopic images delineate the FQ-mediated reduction of lung cancer cell invasion (right-hand graphs).

To exclude the possibility that FQ-mediated inhibition of cancer cell migration was a consequence of its cytotoxic effect, we first defined the appropriate concentration of FQs that showed no adverse effect on cell viability. We evaluated the effect of a concentration range (0–40 µM) of FQs (ciprofloxacin, enrofloxacin, gatifloxacin, moxifloxacin, levofloxacin, and clinafloxacin) on the viability of A549 cells (Figure 1A) and HepG2 cells (Figure S1) using the 3-(4,5-dimethylthiazol-2-yl)-2,5-diphenyltetrazolium bromide (MTT) assay. The results motivated the use of sublethal doses (≤20 μM) of FQs in subsequent experiments. In addition, the MTT and calcein-AM viability assays showed that TGF-β and PMA treatment induced neither cell proliferation nor cell death in A549 cell cultures (Figure S2), suggesting that the TGF-β and PMA-induced increase in MMP-2/9 levels in the culture media was not due to an increase in cell numbers or non-specific release from damaged plasma membranes.

To study the effects of FQs on cancer cell migration and metastasis, we performed wound healing and cell invasion assays. The wound healing assay showed that the mobility of HepG2 cells was enhanced by TGF-β stimulation (Figure 1B), and 20 μM ciprofloxacin significantly decreased the migration ability of A549 cells by 42.4% (*p* ≤ 0.01) compared with TGF-β stimulation (Figure 1C). In the cell invasion assay, treatment with PMA (Figure 1D,E) as well as TGF-β (Figure 1F,G) promoted invasion by 62.4% to 75.6%, respectively, compared with the control; ciprofloxacin and clinafloxacin (20 μM) pre-treatment decreased PMA and TGF-β-induced invasion of A549 cells by 48.2% to 54.7% (*p* ≤ 0.01) (Figure 1D–G). The results revealed that PMA- and TGF-β-induced migration and invasion of A549 cells were significantly suppressed in the FQ pretreatment groups compared with the controls.

### 2.2. FQs Reduced TGF-β-Induced MMP-9 Expression and Secretion

**Figure 2:**
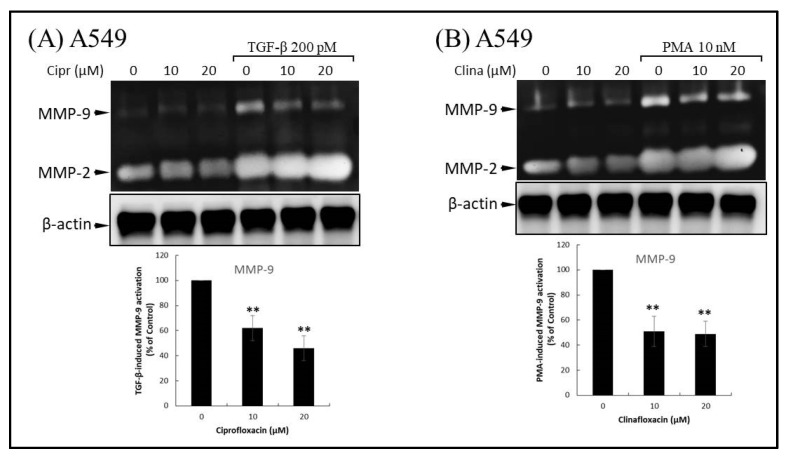
Fluoroquinolones (FQs) inhibit extracellular secretion of matrix metalloproteinase-9 (MMP-9). (**A**) Ciprofloxacin inhibits transforming growth factor beta (TGF-β)-stimulated gelatinolytic activity of MMP-9. A549 cells were pre-treated with ciprofloxacin for 1 h and subsequently treated with TGF-β for an additional 48 h. Conditioned medium was collected for gelatin zymography analysis of gelatinolytic activities. (**B**) Clinafloxacin reduces phorbol 12-myristate 13-acetate (PMA)-induced extracellular secretion of MMP-9. A549 cells were treated with clinafloxacin for 1 h, and then stimulated with 10 nM PMA for 24 h. Representative gelatin zymography images are shown in the upper panel. The MMP-9 activities of the FQ-treated groups were expressed as a percentage of the MMP-9 activity in TGF-β or PMA-treated cells (lower panel). The results are expressed as means ± standard deviation (error bars) of three independent experiments. Statistically significant differences of zymographic activities were indicated as percentage vs. PMA or TGF-β-stimulated control. ** *p* < 0.01 versus PMA or TGF-β-stimulated control experiments.

Various ECM-remodeling enzymes are produced during cancer development and promote cell migration and tumor metastasis. There is overwhelming evidence that MMP-9 promotes cancer cell migration and tumor dissemination. To assess the inhibitory effects of FQs in TGF-β and PMA-induced MMP-9 generation in cells, we used gelatin zymography analysis to examine the effect of ciprofloxacin, levofloxacin, or enrofloxacin on TGF-β and PMA-stimulated MMP-9 activation. A549 cells (10^6^ cells) were preincubated with 10–20 μM ciprofloxacin (Figure 2A) or clinafloxacin (Figure 2B) for 1 h at 37 °C. The cells were stimulated by adding 200 pM TGF-β or 10 nM PMA for 48 h. The ability of TGF-β and PMA to stimulate MMP-9 activation and MMP-9 activity was significantly inhibited by preincubation of A549 with ciprofloxacin and clinafloxacin, respectively. Conversely, MMP-2 expression and activity were detected even in vehicle-treated cells and were not affected by FQ treatment. These results suggest that FQ treatment inhibited TGF-β and that PMA-induced migration may be regulated by the reduction of MMP-9.

**Figure 3:**
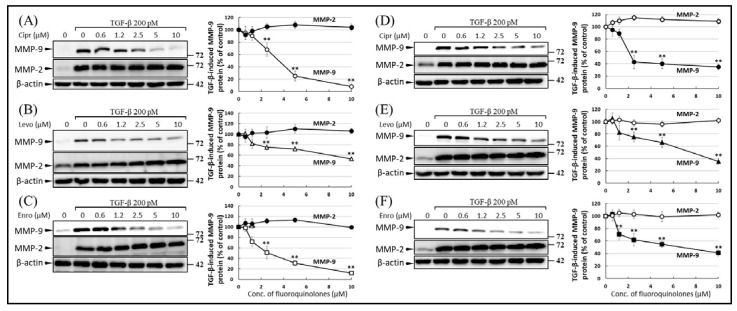
Fluoroquinolones (FQs) inhibit transforming growth factor beta (TGF-β)-induced matrix metalloproteinase-9 (MMP-9) expression in A549 (**A**–**C**) and HepG2 (**D**–**F**) cells. Cells were pre-treated with increasing concentrations of ciprofloxacin (**A**,**D**), levofloxacin (**B**,**E**), and enrofloxacin (**C**,**F**) for 1 h, followed by stimulation with TGF-β for another 48 h; the protein levels in the conditional medium were determined by Western blotting. The upper and lower bands represent MMP-9 (85 kDa) and MMP-2 (70 kDa), respectively. Images shown in (**A**–**F)** are representative of three independent experiments with similar results. The MMP-9 and MMP-2 levels in the right graphs indicated treatment groups were normalized by β-actin and were expressed as a percentage of those in the TGF-β-stimulated control. The differences between treatment groups were considered significant at *p ≤* 0.01 (**; right-hand graphs).

The inhibitory effect of FQs on MMP-9 production was confirmed by Western blot analysis. The MMP-9 band was detectable in the conditioned medium (CM) of TGF-β-stimulated A549 (Figure 3A–C) and HepG2 (Figure 3D–F) cells; TGF-β-induced secretion of MMP-9 was attenuated by treatment with ciprofloxacin (Figure 3A,D), levofloxacin (Figure 3B,E), and enrofloxacin (Figure 3C,F). Furthermore, FQs inhibited the TGF-β-induced production of MMP-9 in a dose-dependent manner (0.6–10 μM). In contrast, FQs did not influence the production of MMP-2 with or without TGF-β stimulation.

TGF-β plays a major role in EMT and regulates the production of ECM proteins such as fibronectin, plasminogen activator inhibitor-1 (PAI-1), and N-cadherin. To investigate whether FQs have the ability to suppress TGF-β-induced MMP-9, we also examined the effects of FQs on TGF-β-induced fibronectin expression. Supplemental Results (Figure S1) showed that FQs did not affect TGF-β-stimulated production of fibronectin, PAI-1, and N-cadherin, indicating that FQs suppress TGF-β-induced MMP-9 via a specific, non-conventional signaling pathway.

**Figure 4:**
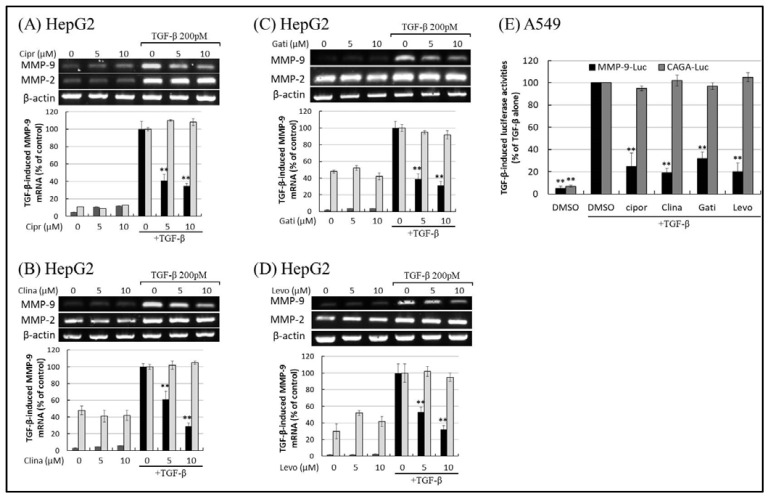
Fluoroquinolones (FQs) inhibit transforming growth factor beta (TGF-β)-induced matrix metalloproteinase-9 (MMP-9) transcriptional activity and gene expression. MMP-9 and MMP-2 gene expression in cells exposed to FQs and TGF-β was detected by reverse transcription polymerase chain reaction (RT-PCR). HepG2 cells were pre-incubated with different concentrations of ciprofloxacin (**A**), clinafloxacin (**B**), gatifloxacin (**C**), and levofloxacin (**D**) for 30 min before stimulation with 200 pM TGF-β. The images shown are representative of three independent experiments with similar results. (**E**) Promoter assays in cells exposed to FQs and TGF-β, similar to RT-PCR. Firefly and renilla luciferase activity were measured in cells co-transfected with MMP-9-Luc, CAGA-Luc, and pRL-CMV and incubated in the presence or absence of FQs and TGF-β. MMP-9 and CAGA firefly luciferase activity was normalized to renilla luciferase activity, and the intensity of the luciferase signal in the treatment groups was expressed as a percentage of that measured in the TGF-β-stimulated experiments. Results are presented as means ± standard deviation (error bars) of three independent experiments. Statistically significant differences (** *p* ≤ 0.001) were observed between cells treated with TGF-β-stimulated controls and those treated with TGF-β and FQs.

To elucidate the effects of FQs on MMP-9 production induced by TGF-β and PMA, we first examined the effects of FQ on the transcriptional activity of the MMP-9 gene. When HepG2 cells were treated with TGF-β, the mRNA expression of MMP-2 and MMP-9 was upregulated (Figure 4A–D). Treatment with ciprofloxacin (Figure 4A), clinafloxacin (Figure 4B), gatifloxacin (Figure 4C), and levofloxacin (Figure 4D) suppressed the transcriptional expression of MMP-9 in a dose-dependent manner. However, the inhibition of MMP-2 was minimal. Treatment with other FQs, such as clinafloxacin and gatifloxacin, also reduced TGF-β-stimulated MMP-9 production, which implied that most FQs share the common ability to suppress MMP-9 production and tumor metastasis [7]. Notably, the decrease in MMP-9 mRNA levels was not due to decreased MMP-9 mRNA stability, because the FQs used in this study did not significantly change the MMP-9 mRNA decay rate, based on the mRNA stability assay using actinomycin D (Figure S3).

Smad3/Smad4 binding sequences, termed CAGA boxes, confer TGF-β stimulation to a heterologous promoter–reporter construct. TGF-β regulates the expression of target genes via activation of the CAGA box. Therefore, we tested the effects of FQ on the activation of the CAGA box and MMP-9 promoter by TGF-β. Reporter assays showed that TGF-β significantly induced activation of the CAGA box (CAGA-Luc) and MMP-9 (MMP-9-Luc) promoters, respectively. Ciprofloxacin, clinafloxacin, gatifloxacin, and levofloxacin blocked the activation of MMP-9 responsive element (Figure 4E) without affecting CAGA box activity and other TGF-β-responsive elements (SBE4; Figure S4). As shown in Figure 2 and Figure 3, the expression of MMP-9 levels and activities mirrored MMP-9 promoter activities. This suppressive effect of FQs on MMP-9 was not attributed to non-specific effects on transcription and/or translation because luciferase activity driven by the classical (Smad-mediated) TGF-β signaling was not affected by treatment with FQs (Figure 4E).

### 2.3. FQs Inhibited Activation of TGF-β-Dependent p38, but Not Smad2/3

**Figure 5:**
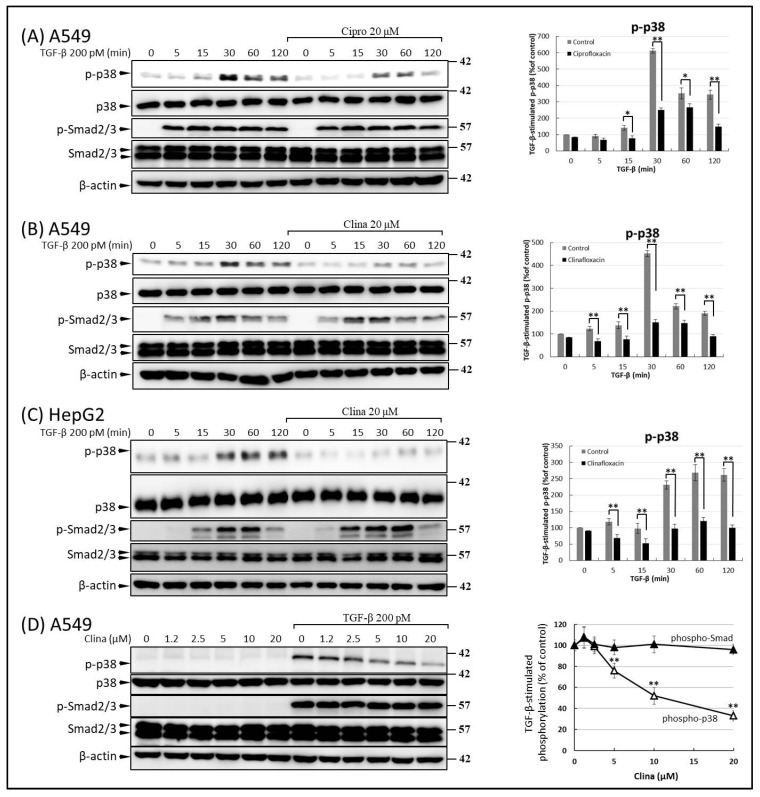
Transforming growth factor beta (TGF-β)-stimulated activation of p38, but not of Smad, was inhibited by fluoroquinolones (FQs). Cultured A549 and HepG2 cells were serum-deprived for 24 h before growth factor stimulation. A549 (**A**–**D**) and HepG2 (**C**) cells were incubated in the presence of 20 μM ciprofloxacin (**A**) or clinafloxacin (**B**,**C**) for 30 min before incubation with 200 pM TGF-β for the indicated times. Smad2/3 and p38 phosphorylations were determined by Western blotting. Phosphorylated protein levels of the indicated experimental groups were quantified as a percentage relative to the level of the control experiments. Data are representative of at least three independent experiments (right-hand panel; * *p* ≤ 0.05, ** *p* ≤ 0.01). (**D**) Because the maximal stimulation of p38 activation by TGF-β is approximately 30 min, A549 cells were treated with 200 pM TGF-β in the absence or presence of the indicated concentrations of clinafloxacin for 30 min, and Smad2/3 and p38 phosphorylation were determined by Western blotting as described in (**A**). Phosphorylated protein levels of the indicated experimental groups were quantified as a percentage relative to the level of TGF-β-treated cells (right-hand graphs; ** *p* ≤ 0.01).

Phosphorylation of Smad proteins plays a central role in TGF-β signaling. To elucidate the mechanisms underlying the suppressive effects of FQs on the TGF-β pathways, we tested the effects of FQs on the phosphorylation of Smad proteins [16]. When cells were treated with TGF-β, phosphorylation of Smad2 and Smad3 (p-Smad2/3) was observed. The TGF-β-inducible phosphorylation of Smad was not suppressed by treatment with ciprofloxacin and clinafloxacin (Figure 5A−D). Since our data showed that FQs suppressed MMP-9 without affecting MMP-2 (Figure 2, Figure 3 and Figure 4) and considering that p38 activation is exclusively responsible for MMP-9 production without affecting MMP-2 [35], we examined whether FQ treatment inhibits TGF-β-stimulated p38 phosphorylation in A549 cells. The temporal profile of TGF-β-induced p38 phosphorylation showed that 30 min of TGF-β treatment resulted in the maximum phosphorylation level of p38 (Figure 5A–C). In A549 (Figure 5A,B) and HepG2 (Figure 5C) cells, pre-treatment with ciprofloxacin and clinafloxacin ameliorated the phosphorylation (Figure 5A–C) at all time-points. The inhibitory effects of FQs on p38 were not specific to A549 cells; similar effects were also observed in HepG2 cells (Figure 5C). In addition, TGF-β-stimulated p38 phosphorylation was attenuated by clinafloxacin in a dose-dependent manner (Figure 5D).

**Figure 6:**
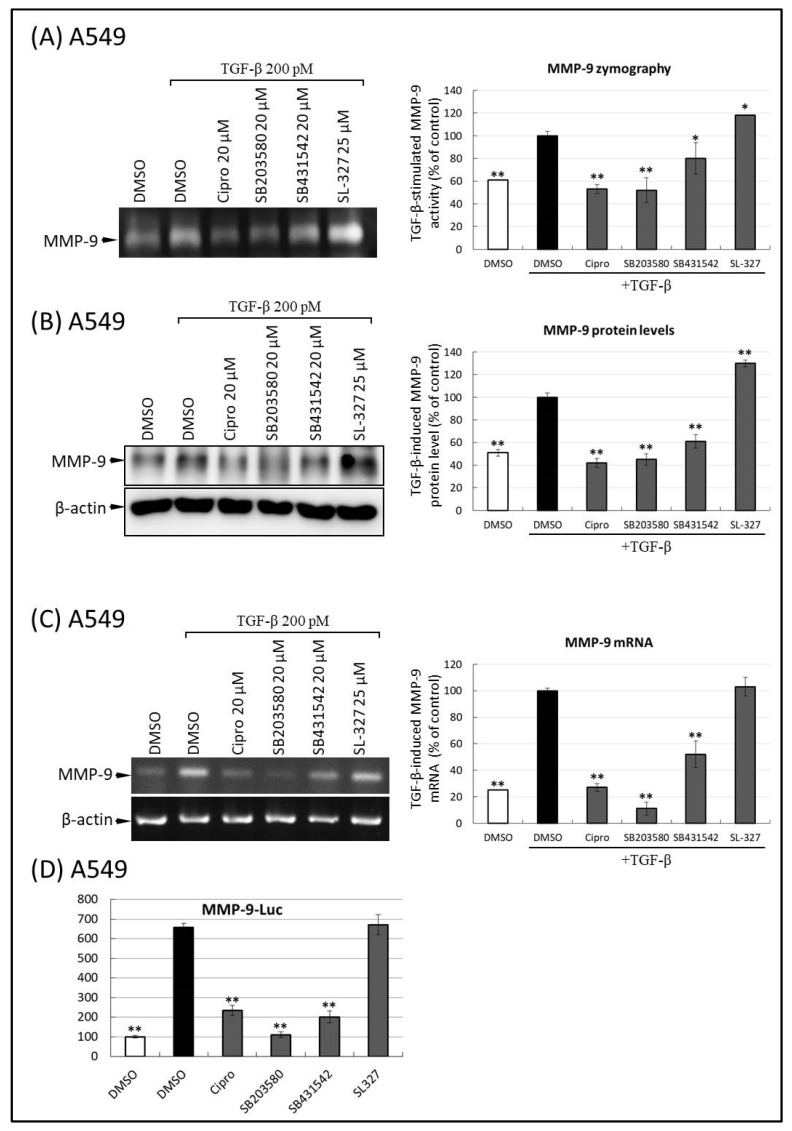
Ciprofloxacin, p38 inhibitor, and transforming growth factor beta (TGF-β) receptor-I inhibitor, but not MEK inhibitor, inhibit matrix metalloproteinase-9 (MMP-9) activity, secretion, and transcriptional activity. (**A**) Ciprofloxacin, p38 inhibitor (SB203580), and TGF-β inhibitor (SB431542) inhibit TGF-β-stimulated gelatinolytic activity of MMP-9 in A549 cells. The conditioned mediums from a similar treatment described in Figure 2A were subjected to gelatin zymography analysis of MMP-9. The conditional mediums from chemical-treated cells were subjected to zymographic analysis of MMP-9. An equal amount of protein loading was verified using a bicinchoninic acid protein assay; the right-hand graph illustrates quantitative analysis (mean ± standard deviation [SD]) from three independent experiments (compare with TGF-β treatment alone); * *p* ≤ 0.05, ** *p* ≤ 0.01. (**B**) Ciprofloxacin, SB203580, and SB431542 prevent TGF-β-induced MMP-9 protein expression in A549. The samples from a similar treatment described in Figure 3 were subjected to immunoblot analysis of MMP-9 and β-actin. An equal amount of protein loading was verified using β-actin; the right-hand graph illustrates quantitative analyses of enhanced chemiluminescence (ECL) (mean ± SD) from three independent experiments (compared with TGF-β treatment alone); ** *p* ≤ 0.01. (**C**) Ciprofloxacin, SB203580, and SB431542 reduced TGF-β-stimulated MMP-9 mRNA transcript production in A549 cells. A549 cells were pre-treated with the indicated concentrations of ciprofloxacin, SB203580, SB431542, and SL-327 for 30 min and subjected to TGF-β (200 pM) stimulation for an additional 12 h. The transcripts were quantified using TaqMan PCR, normalized to β-actin levels, and expressed as a percentage (mean ± SD) of that of the TGF-β treatment group. ** *p* < 0.001 vs. the TGF-β-treated group. (**D**) Ciprofloxacin, SB203580, and SB431542 inhibited TGF-β-induced MMP-9 promoter activation in A549 cells. The experimental condition and materials of promoter assays in cells exposed to FQs and TGF-β were similar to the experiment described in Figure 4D. Statistically significant differences (** *p* ≤ 0.001) were observed between cells treated with TGF-β only and those treated with TGF-β, FQ, and inhibitors. All images shown in (**A**–**C**) were representative of three independent experiments with similar results.

It was previously established that the p38 pathway is involved in TGF-β-induced MMP-9 secretion in epithelial cells. To further understand the role of p38 in FQ-inhibited MMP-9 production, we tested whether SB203580, a selective and well-validated inhibitor of p38 MAP kinase, decreased TGF-β-induced MMP-2/9 upregulation in A549 cells. As depicted in Figure 5, TGF-β treatment of A549 cells induced the activation of both MMP-9 activity (Figure 6A) and protein levels in CM (Figure 6B). When these cells were preincubated with ciprofloxacin, SB203580, SB431542, or SL-327, TGF-β-stimulated MMP-9 activation and production were inhibited by ciprofloxacin, p38, and ALK5 inhibitors. In contrast, the secretion and activation of MMP-9 by TGF-β was unaffected by pretreatment with the MEK1/2 inhibitor, SL-327 (Figure 6A,B). To test whether the p38 pathway mediates the inhibitory effect of FQ on TGF-β-induced MMP-9 transcriptional activity, quantitative reverse transcription polymerase chain reaction (RT-PCR) analysis was performed. As shown in Figure 6C, co-treatment of A549 cells with ciprofloxacin, SB203580, and SB431542 inhibited the upregulation of MMP-9 mRNA by TGF-β. The role of the p38 signaling pathway in FQ-attenuated MMP-9 was also evaluated by measuring MMP-9 promoter activity. To do this, the A549 cells transiently expressing an MMP-9-Luc were pre-treated with ciprofloxacin, SB203580, SB431542, and SL-327, respectively. As shown in Figure 6D, TGF-β-induced MMP-9 promoter activation was almost completely blocked in ciprofloxacin-, SB203580, and SB431542 treated cells. However, no inhibitory effect was observed in SL-327-treated cells.

**Figure 7:**
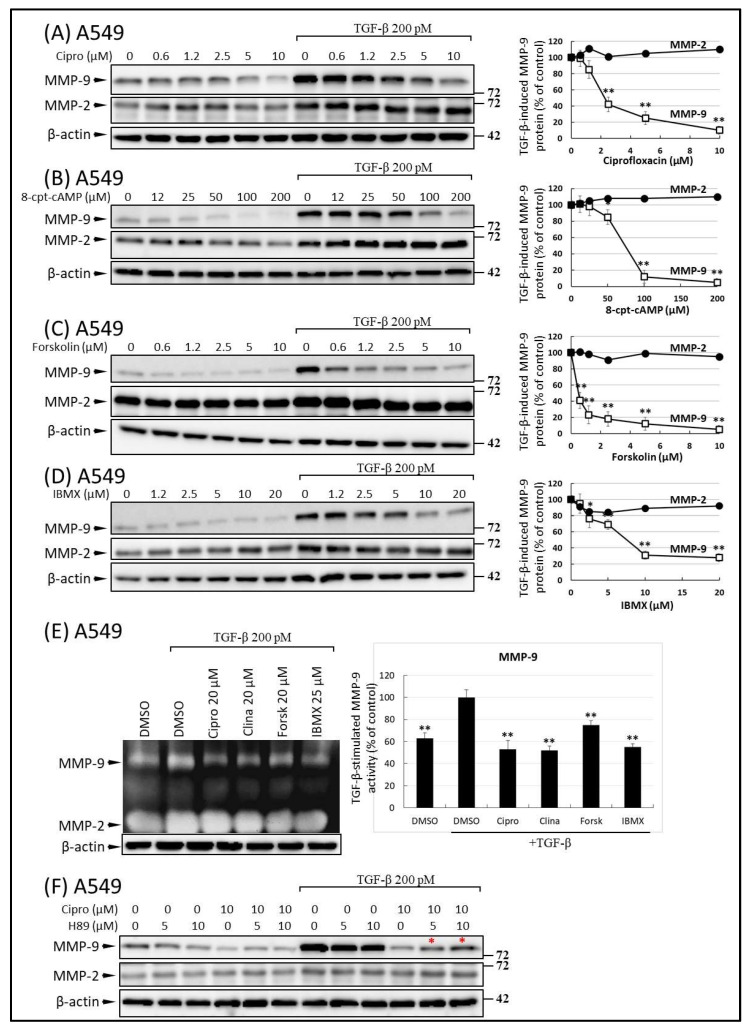
Effects of ciprofloxacin and cyclic AMP mimetics on transforming growth factor beta (TGF-β)-induced secretion of matrix metalloproteinase-9 (MMP-9). Cells were preincubated with increasing concentrations of (**A**) ciprofloxacin, (**B**) 8-cpt-cAMP, (**C**) forskolin, and (**D**) isobutyryl methylxanthine (IBMX) prior to the addition of 200 pM TGF-β for 48 h. MMP-9 protein levels were determined in the culture medium by Western blot analysis. For details see the legend to Figure 3 and Materials and Methods (Section 4.5). Data are presented as mean ± standard deviation (*n* = 3). * *p* ≤ 0.01, ** *p* ≤ 0.001 vs. control (TGF-β stimulated cells). (**E**) Inhibitory effect of clinafloxacin and cAMP on MMP-9 activity in TGF-β stimulated A549 cells (*n* = 3). Cells were incubated with ciprofloxacin, clinafloxacin, forskolin, and IBMX at the indicated concentrations prior to addition of 200 pM TGF-β for 48 h. MMP-9 activities were determined in the culture medium by gelatin zymography. (**F**) H89, a protein kinase A inhibitor blocks the inhibitory effect of MMP-9 on ciprofloxacin release. A549 cells were incubated with or without H89 (5 and 10 μM) for 15 min, then with or without ciprofloxacin (10 μM) for 5 min, and then continued with or without TGF-β (200 pM) for 48 h. The conditioned medium was analyzed by Western blotting and densitometry. (**F**) A representative image revealed that H89 alone or in combination with TGF-β did not modify MMP-9 secretion; it prevented the ciprofloxacin-mediated inhibition of MMP-9 secretion (indicated by red asterisk).

The activation of cAMP has been linked to impaired wound healing and reduced migratory abilities of cancer cells via the inhibition of the p38 pathway [11,12,13,14]. It was previously established that the accumulation of cAMP and subsequent PKA activation are crucial steps that drive the anti-migratory signaling of FQs [20,22,36]. It is tempting to speculate that FQ-induced cAMP may contribute to the inhibition of MMP-9 production and motility in malignant tumor cells. To examine the role of cAMP in the modulation of MMP-9 synthesis by FQ treatment, we examined the effects of various concentrations of the cAMP isomers 8-cpt-cAMP (an agonist), an adenylyl cyclase activator (forskolin), and the phosphodiesterase inhibitor, 3-isobutyl-1-methyl-xanthine (IBMX), on the levels of MMP-9 and MMP-2. Forskolin (Figure 7C), 8-cpt-cAMP (Figure 7B), and IBMX (Figure 7D), similar to ciprofloxacin (Figure 7A), inhibited the TGF-β-induced production of MMP-9. Similarly, MMP-2, which is insensitive to FQ treatment, was also unaffected by pretreatment with cAMP-induction drugs (Figure 7B–D). For additional confirmation of the effect of cAMP on MMP-9 activity, we investigated the effects of FQ and cAMP-induction drugs on TGF-β-mediated MMP-9 activation; TGF-β treatment of A549 cells induced rapid activation of both MMP-9 and MMP-2 (Figure 7E). However, when these cells were preincubated with ciprofloxacin, clinafloxacin, forskolin, or IBMX, only MMP-9 activity was inhibited (Figure 7E). 

Next, to examine whether the inhibitory effect of FQ on the TGF-β-induced production of MMP-9 occurs via PKA, we used H-89, an inhibitor with a high specificity for PKA [36]. Starved A549 cells were incubated with or without H-89 (5 and 10 μM) for 30 min and then incubated with ciprofloxacin for 30 min. After the treatments, A549 cells were stimulated with TGF-β (200 pM). Cell culture supernatants were collected 48 h after the initiation of TGF-β stimulation. Notably, pretreatment with H-89 (5 and 10 μM) partially reversed the inhibitory effects of ciprofloxacin on inducing MMP-9 secretion in a dose-dependent manner (Figure 7F).

## 3. Discussion

Previous and current studies demonstrate an effect of FQs on the expression level of MMP-9 [15], suggesting that FQs could be a potential therapeutic agent for ameliorating cancer development and dissemination. We also revealed that FQ inhibits MMP-9 expression via p38 and cAMP-dependent pathways in TGF-β-and PMA-stimulated A549 cells. MMPs are also involved in tumor invasion and metastasis [37]. In contrast to constitutively expressed MMP-2, MMP-9 is inducible, and represents one of the most important proteinases that mediates tumor progression in human lung cancer [38], gliomas [39], pancreatic cancer [40], head and neck cancer [41], prostate cancer [42], gastric cancer [43], and breast cancer [44]. Furthermore, unlike MMP-2, MMP-9 activity is not detectable in normal adult tissue [45]. MMP-9 expression is considered a major prerequisite for tumor invasion. Inhibition of MMP-9 activity by specific drugs or antisense oligonucleotide techniques impairs tumor progression and invasion. The expression of MMP-9 is tightly and specifically regulated at the transcriptional level via multiple signaling pathways. In particular, TGF-β, PMA (PKC activator), tumor necrosis factor alpha (TNF-α), IL-1β, and IL-6 stimulate MMP-9 expression in a dose-dependent manner. Since the MMP-9 gene is inducible in cancer cells and TGF-β-dependent induction of MMP-9 promoter is relevant in tumor cells displaying invasive potential, we used TGF-β and PMA as inducers to study the effects of FQs on MMP-9 activity.

Given that MMP-9 and MMP-2 are both produced in response to TGF-β and PMA and have certain overlapping and synergistic activities [3,13,39,46], we compared the effects of the five FQs on both MMP-9 and MMP-2 production. Our results (Figure 2 and Figure 3) show that under physiologically relevant concentrations (up to 10 μM), the various FQs reduced, to a similar degree, the extracellular production of MMP-9 by TGF-β or PMA-stimulated A549 cells. However, the FQs used in this study did not affect TGF-β or PMA-stimulated MMP-2 levels in the cells, suggesting that the effects of FQs on cell-associated MMP-9 and MMP-2 production are different.

**Figure 8:**
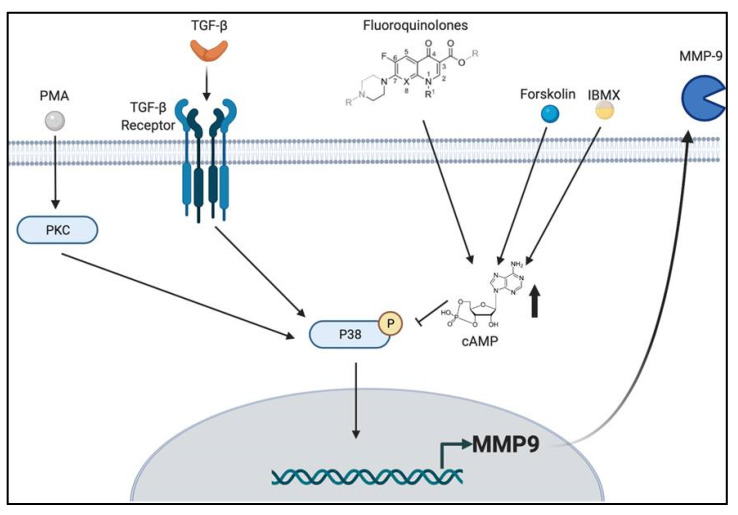
The model by which fluoroquinolones (FQs) inhibit matrix metalloproteinase-9 (MMP-9) production by modulating p38 and cyclic AMP (cAMP) signaling. In epithelial cells, transforming growth factor beta (TGF-β) promotes invasion and metastasis (100% growth inhibition at 200 pM) by stimulating the p38-mediated signaling cascade in concert with canonical TGF-β signaling mediated by TGF-β receptor I (TβR-I) and TβR-II (TβR-I/TβR-II/Smad-2/3/4). It has been recognized that FQs exert their anti-inflammation by producing intracellular cAMP, and we discovered that FQs antagonize TGF-β and phorbol 12-myristate 13-acetate (PMA)-induced MMP-9 production by suppressing phosphorylation of p38 via cAMP production or via their components.

A prominent finding of this study is that the inhibition of MMP-9 by FQ does not depend on the activation of a single signaling pathway, but on at least two signaling pathways, namely, the p38 and cAMP pathways (Figure 8). Although each single pathway was previously shown to be involved in MMP-9 production in response to different stimuli [23,24,36,46,47,48,49,50], our data presented here should increase the interest in FQs as anticancer agents targeting MMP-9.

TGF-β signaling is initiated by successive activation of the TβR-II and TβR-I kinases, followed by activation of canonical (Smad-mediated) or non-canonical pathways [51]. p38 MAP kinase is one of the major non-canonical pathways for TGF-β, but TGF-β is also known to activate other MAP kinases, such as ERK and c-Jun N-terminal kinase (JNK) [51]. Because the promoter region of the MMP-9 gene contains AP-1 and NF-κB activating sites [52], ERK1/2 and JNK are also implicated in the signal transduction cascade in the upregulation of MMP-9 [52,53]. The inhibitors of p38, ERK, and JNK used in this study are reported to be highly specific [34,54,55,56,57], and no obvious phenotypic changes in the cells were observed during the experiments. Among these, the p38 inhibitor showed potent inhibitory effects on MMP-9 production, suggesting that p38 kinases may be more critical than ERK and JNK kinase in TGF-β-mediated MMP-9 induction in lung cancer cells. In addition, previous reports have also supported the notion that TGF-β-induced MMP-9 expression is mediated through the activation of p38 MAPK, but not ERK1/2, in MCF10A human breast epithelial cells [47] and in human glioblastoma cells [46]. The different results may be due to the different cell types and experimental conditions.

To explain the observation of an FQ-induced decrease in extracellular MMP-9 production, we investigated the possible involvement of cAMP, a second messenger known to act at various levels of intracellular signal transduction. We demonstrated that FQs increased intracellular cAMP concentrations in both resting and TGF-β-stimulated cells. 8-CPT-cAMP (an active analog of cAMP), forskolin, and IBMX markedly inhibited TGF-β and PMA-induced secretion of MMP-9 (Figure 7B–E). In contrast, N-[2-p-bromocinnamylamino-ethyl]-5-isoquinolinesulfonamide (H89), a competitive inhibitor (antagonist) of cAMP that binds to and inactivates PKA, prevents the inhibitory effects of FQ in TGF-β-stimulated synthesis of MMP-9 (Figure 7F), further confirming the role of the cAMP-mediated pathway in the FQ-mediated regulation of MMP-9 secretion. In addition to inhibiting MMP-9 production, it has been reported that FQs or cAMP promoting agents such as prostaglandin E2, dibutyryl cAMP, or phosphodiesterase inhibitors reduce the expression levels of inflammatory cytokines, including TNF-α, IL-1, IL-6, and IL-8 [19,36,58,59]. Since TNF-α and IL-1 are strong inducers of MMP-9 synthesis, it is likely that FQs and other cAMP-promoting agents repress MMP-9 production by reducing the levels of the responsible cytokines. Defining the biochemical links between MMP-9 production and the secondary functions of FQs could reveal new mechanisms of signal-driven antitumor action.

Interference with cell receptors such as the TGF-β receptors, alone or in concert with the various stimuli, could explain the observations relating to the inhibitory effects of FQs. However, at present, there is no evidence of any direct interaction or molecular interference of FQs with TGF-β receptors or downstream signaling molecules associated with signal transduction. Similarly, the direct effect of quinolone antibiotics on various kinases may also explain many of the reported modulatory effects of FQs. Indeed, some preliminary reports indicated that quinolone antibiotics may have such an effect on PKC and tyrosine kinase in PMA-and *N*-formyl-met-leu-phe (fMLP)-stimulated cells; however, other studies using various agonists and antagonists of the aforementioned kinases have not shown any direct effects of quinolone antibiotics on these molecules [19,20,60]. Therefore, the direct molecular targets of FQs in anticancer and immunomodulatory activities remain unclear.

Some FQs, including ciprofloxacin, enrofloxacin, moxifloxacin, and gatifloxacin, have been shown to induce DNA double-strand breaks via topoisomerase II (topo II) inhibition, stabilize cleavage complex formation, and further activate the p53/Bax/Bcl-2 signaling pathway, although these are still matters of debate [19,20,61]. FQs can also inhibit the secretion of inflammatory cytokines by inhibiting phosphodiesterase, leading to the accumulation of intracellular cAMP. In a physiologically achievable concentration, ciprofloxacin was reported as a phosphodiesterase inhibitor, resulting in significant inhibition of TNF-α and IL-6 production from the stimulated cells; the authors indicated that the effects of ciprofloxacin were similar to those of cAMP agonists [22,62]. However, the molecular mechanism of the proposed phosphodiesterase inhibition exerted by FQ is currently unknown and should be studied in detail. Our present data indicate that the induction of intracellular cAMP and inhibition of p38 phosphorylation could be the potential anti-metastatic mechanisms of the FQs used in this study.

This study has limitations in that we did not clarify whether the FQs affect other cell signaling pathways, as reported for other macrolides and FQs, which downregulate the expression of nuclear factor-1 of activated T cells, NF-κB, or AP-1 [19,54]. Therefore, further studies are necessary. Our finding that multiple signaling pathways are involved in the inhibition of MMP-9 by TGF-β (and possibly stimulation by PMA and other growth factors) has expanded our knowledge on the regulation of this important molecule involved in tumor invasion and metastasis. In our ongoing work, we are targeting more signaling pathways that specifically repress the function of MMP-9. Indeed, the applications of ciprofloxacin, moxifloxacin, and levofloxacin dramatically inhibited lung cancer cell invasion in vitro (Figure 1). These data provide insights and more options for future clinical interventions targeting TGF-β-induced MMP-9 upregulation and cancer invasion.

Previous studies revealed that increased expression of MMP-9 is associated with poor prognosis in patients with several types of cancer [1,41,42,43,55] and increased invasion and metastasis of cancer cells [4,8,41]. Considering the role of the enzyme, the use of MMP-9 inhibitors may have therapeutic potential, but unfortunately, the lack of specificity of the inhibitors often leads to the inhibition of off-target MMPs, resulting in unacceptable side effects. In previous studies, the use of these inhibitors has resulted in side effects owing to the inhibition of closely related enzymes, such as a disintegrin and metalloproteinases (ADAMs) and ADAMs with a thrombospondin domain (ADAMTSs); they are also marked by poor pharmacokinetic profiles, lack of specificity, and toxicity. For this reason, new technologies, such as expression microarrays, degradomic approaches, and RNA-Seq methods for gene expression profiling, will provide a broader view of the underlying pathology as well as the role of MMP-9 inhibitors and other natural inhibitors. This approach would help to identify new treatment options.

Selective control of MMP-9 activity may be achieved by exosite interactions (allosteric inhibitors), transcriptional regulation, repurposing of known drugs, and the discovery of highly selective natural compounds. Although investigations into the role of FQs in curing MMP-9 related diseases are in the preliminary stages, it is worth noting that tetracycline and its derivatives have been studied and applied to lower the levels of MMP-9 secretion and inhibit MMP-9 activity. Among these, doxycycline and minocycline have been thoroughly evaluated. Furthermore, some advances have been made in this treatment approach by preventing the upstream pathway from indirectly targeting proMMP-9 activation. For example, the plasminogen/MMP-9 cascade is a promising target for regulating inflammatory responses and development of abdominal aortic aneurysms (AAAs) [55]. Targeting the protease nexin 1/protease/low density lipoprotein receptor-related protein 1 (LRP-1) pathway also reduces the secretion of MMP-9 [56]. A wealth of evidence suggests that MMPs other than MMP-9 may be involved in tumor dissemination and metastasis. Cancer can produce a wide spectrum of proteases, including MMP-1, MMP-3, MMP-7, MMP-8, and MMP-14 [57]. It would be interesting for future studies to examine whether FQs inhibit the production of other MMPs in tumor cells in response to TGF-β stimulation and during metastasis.

Considering that all of the FQs in this study have been determined to be anti-migratory agents, the synthesis of new FQ derivatives, based on their activity against MMP-9 production, could be pursued for the development of new antitumor agents. In this study, the inhibition of MMP-9 synthesis was shown to be directly proportional to the anti-migratory activity of the drug. Therefore, screening of new FQs with more potent MMP-9 targeting activity seems to be a feasible approach to develop a potential antitumor agent. The pharmacokinetic and toxicological features of FQs, which have been well investigated for their clinical use as antibacterial agents, will provide useful information on the therapeutic application of FQs as antitumor agents in combination with other drugs.

In summary, FQs are a promising new class of antitumor agents that target MMP-9. Owing to the despairingly poor prognosis for conventional procedural patients with cancer, as well as the soaring costs and lengthy development phase required for advancement in new medications, our findings offer mechanism-motivated prospects to repurpose FQ derivatives as anticancer agents due to several advantages: (1) from a molecular perspective, the ability of FQ to activate the antitumoral p53 pathway by affecting DNA repair beyond a barricade, alleviating the adverse effect of other topoisomerase inhibitors; (2) FQs effectively suppress both constitutive and stimulated inflammatory cytokines that are major contributing factors in cancer development and malignancy; (3) from a clinical standpoint, antibiotics are already commonly used for infection prophylaxis during chemotherapy. In addition, the adverse effects of FQs are mild or do not synergize with the main side effects of chemotherapy; and (4) the skyrocketing cost of new drug development provides an economic basis for the repurposing of FQs for adjuvant therapy if the combination becomes available for cancer patients.

## 4. Materials and Methods

### 4.1. Cell Culture

The human lung cancer cell line (A549) and hepatocellular carcinoma cell line, (HepG2) were gifts from Dr. Ming-Hung Tai of National Sun Yat-Sen University. The A549 and HepG2 cells were grown in Dulbecco’s modified Eagle’s medium (DMEM) supplemented with 10% fetal bovine serum (FBS), 100 U/mL penicillin, and 100 μg/μL streptomycin (Biochrom AG, Berlin, Germany). Cell cultures were grown to confluence and maintained in a humidified atmosphere at 37 °C and 5% CO_2_. The A549 and HepG2 cells were incubated with FBS-free medium for 24 h prior to the experiments; FQs were then added to achieve a final concentration in the range of 1–20 μM.

### 4.2. Reagents and Antibodies

The FQs (ofloxacin, levofloxacin, ciprofloxacin, moxifloxacin, gatifloxacin, clinafloxacin, nadifloxacin, sarafloxacin, tosufloxacin, and enrofloxacin) used in this study were purchased from Cayman Chemical (Ann Arbor, MI, USA). The FQ stock solution (20 mM) was prepared in DMSO. The final concentration of DMSO in all experiments was lower than 0.1%, with no effects on cell function. TGF-β was obtained from PeproTech (Rocky Hill, NJ, USA). TRIzol reagent was purchased from Invitrogen (Carlsbad, CA, USA). Moloney murine leukemia virus reverse transcriptase was obtained from Promega (Madison, WI, USA). The MMP-9, fibronectin, CAGA-luciferase, and SBE4-luciferase reporter plasmids were constructed as described previously [58,59]. Forskolin, IBMX, PMA, 8-CPT-cAMP, H89, MTT, DMEM, trichloroacetic acid (TCA), aprotinin, phenylmethanesulfonyl fluoride (PMSF), and peroxidase-conjugated anti-rabbit IgG were purchased from Sigma-Aldrich (St. Louis, MO, USA). A pre-stained protein marker (125, 93, 72, 57, 42, 31, 24, and 15 kDa) was obtained from GeneDireX (Carlsbad, CA, USA). Information on the antibodies used in Western blot analysis is listed in Supplementary Table S1. The bicinchoninic acid (BCA) assay kit and Lipofectamine 3000 reagent were supplied by Thermo Fisher Scientific (Waltham, MA, USA). Polyvinylidene fluoride (PVDF) membranes were purchased from PALL Corporation (Port Washington, NY, USA).

### 4.3. Cell Viability and Toxicity Assays 

The effect of FQs on cell viability was determined using the MTT assay, according to the protocol described in our previous work [60]. The A549 and HepG2 cells were grown in 96-well plates at a density of 5000 cells/well, 24 h prior to the experiment. During the experiment, the cells were transferred to low serum (0.5% FBS) media and were treated with increasing concentrations of FQs for 48 h. Subsequently, 50 μL MTT solution (1 mg/mL) was added to each well and incubated for 4 h at 37 °C. Finally, formazan crystals were solubilized in 200 μL DMSO, and the absorbance was measured at λ = 560 nm using a microplate reader (San Jose, CA, USA). The experiments were performed three times in triplicate.

### 4.4. Preparation of CM

CM was prepared by the addition of 10 μg/mL aprotinin and 0.5 μM PMSF, precipitated using 10% TCA (Sigma-Aldrich T9159) after the cells had been spun out and stored at –80 °C. An alternative method using a filter unit with a molecular exclusion of 30,000 Da was used for CM inspissation (Millipore Corporation, Billerica, MA, USA). The protein concentration of CM was determined using a BCA assay and normalized by adding various volumes of radioimmunoprecipitation assay buffer. CM samples containing 30 μg of protein were subjected to sodium dodecyl sulfate–polyacrylamide gel electrophoresis (SDS-PAGE) under reducing conditions and were further analyzed by Western blotting.

### 4.5. Western Blot Analysis

Western blot analysis was performed according to the protocol described in our previous study [61]. Cells were preincubated in serum-free medium for 3 h prior to the experiment. A549 and HepG2 cells were incubated with FQs for 1 h and incubated at 37 °C for 48 h in the absence or presence of 200 pM of TGF-β or 10 nM PMA. For Western blot analysis, proteins in conditioned medium and cytosolic fractions were homogenized in SDS–PAGE sample buffer (100 mM Tris-HCl [pH 6.8], 2% SDS, 20% glycerol, 1 mM dithiothreitol, and 0.01% bromophenol blue) containing sodium orthovanadate was resolved by 10% SDS-PAGE. After electrophoresis and electrophoretic transfer of proteins to PVDF membranes, the membranes were blocked with 5% non-fat milk in Tris-buffered saline (pH 7.4) containing 0.1% Tween (TBST) for 1 h. Membranes were probed with primary antibodies at 4 °C for 24 h and further incubated with a secondary antibody, horseradish peroxidase-linked anti-rabbit IgG (GE Healthcare UK Ltd., Buckinghamshire, UK) for 1 h at room temperature. The blots were washed three times in TBST and visualized using enhanced chemiluminescence (Amersham ECL Plus Western Blot Detection System; GE Healthcare). The specific bands were photographed using an ImageQuant LAS 4000 (GE Healthcare Inc., Chicago, IL, USA). Relative density values were determined by densitometric analysis using ImageQuant (GE Healthcare Inc., Chicago, IL, USA).

### 4.6. Gelatin Zymography

The cells were serum-starved in DMEM and treated with or without 200 pM of TGF-β or 10 nM of PMA for 48 h in the absence or presence of FQs. The protein concentration of conditioned medium (CM) was determined using a BCA assay and normalized by adding various volumes of radioimmunoprecipitation assay buffer. CM samples containing 30 μg of protein were separated by SDS-PAGE under non-reducing conditions. Gelatin (2 g/L) was added to the gel for gelatin zymography. The volume of media loaded onto the gel was adjusted for equivalent protein levels. After electrophoresis, the gels were washed twice in 50 mM Tris-HCl containing 25 mL/L of Triton X-100 for 30 min to completely eliminate SDS. The gels were then rinsed twice in zymogen activation buffer (50 mM Tris-HCl, 0.2 g/L Brij 35, 5 mM CaCl_2_ and 0.2 mM NaCl for gelatin zymography and 30 mM Tris [pH 7.4], and 0.2 g/L NaN_3_) and incubated for 48 h at 37 °C in the same buffer. After incubation, the gels were stained for 2 h with 2.5 g/L Coomassie Brilliant Blue R-250 solution and destained with destaining buffer (20% methanol, 10% acetic acid, and 70% water).

### 4.7. Wound Healing Assay

Cells were seeded in a 12-well cluster tissue culture plate and allowed to grow to confluence in growth medium. Subsequently, a cell-free zone was manually created by scratching the cell monolayers with a 10-μL pipette tip. The wounded cell monolayers were washed three times with phosphate-buffered saline and incubated at 37 °C for 24 h in DMEM containing 0.1% FBS containing 200 pM TGF-β, alone or in combination with 10 μM of the FQ under investigation. Five scratched fields in individual wells were randomly chosen, and the images were captured using a CoolSNAP HQ2 Monochrome camera (Photometrics, Tucson, AZ, USA) under an Axio Observer Z1 inverted microscope (Zeiss, Oberkochen, Germany), and time-lapse images were taken within a live-cell atmosphere chamber (Pecon, Oberkochen, Germany) at 37 °C and 5% CO_2_.

### 4.8. Cell Invasion Assay

Cells were detached from the culture plates and resuspended in serum-free medium. A cell suspension containing 20,000 cells was added to the upper well of the transwell inserts (pore size: 8 μm; BD Biosciences, San Jose, CA, USA). In the lower well, a complete growth medium (700 μL) was used as a chemoattractant. Cells that did not migrate to the lower compartment were removed using a cotton swab. The cells on the lower surface of the membrane were stained with crystal violet for 10 min and then air-dried. Inserts were mounted on glass slides, and five random fields at a magnification of 20× were counted per sample. 

### 4.9. Determination of Intracellular cAMP Level

The cAMP concentration was measured using a Cyclic AMP Competitive Enzyme Immunoassay Kit (R&D Systems, Minneapolis, MN, USA). Briefly, cells were lysed in 0.1 M HCl to inhibit phosphodiesterase activity. Following neutralization and dilution, a fixed amount of cAMP direct conjugate was added to compete with cAMP in the cell lysates for sites on a rabbit polyclonal antibody immobilized on a 96-well plate. After adding cAMP antibody to each well, the plate was incubated at room temperature for 2 h. Following a wash to remove excess monoclonal antibody, a fixed amount of horseradish peroxidase-labeled cAMP was applied to compete with cAMP present in a sample for sites on the monoclonal antibody. This was followed by another wash to remove excess conjugate and unbound samples. A substrate solution was added to the wells to determine bound enzyme activity. The color development was stopped, and the absorbance was read at 450 nm. The intensity of the color was inversely proportional to the concentration of cAMP in the cell lysates.

### 4.10. Luciferase Assay

Luciferase reporter assays for HepG2 or MLE cells were performed as described in our previous report. MMP-9-Luc and Renilla luciferase (for data normalization) plasmids were co-transfected into HepG2 cells using electroporation (Gene Pulser Xcell; Bio-Rad Laboratories, Hercules, CA, USA). Ten hours after transfection, the cells were pre-incubated with FQs 6 h before the addition of TGF-β (200 pM). In a similar experiment, HepG2 cells stably expressing the CAGA-Luc promoter plasmid were used. Cells were treated with increasing amounts of FQ (1.2–20 μM) for 6 h, and the data were compared to cells treated with DMSO as a control. Plates were then stimulated with or without TGF-β for 20–22 h. Cells were then solubilized in lysis buffer (25 mM Tris-phosphate, 2 mM 1,2-diaminocyclohexane-N,N,N′,N′-tetraacetic acid, 2 mM dithiothreitol, 10% glycerol, and 0.5% Triton X-100), and luciferase activities were determined using the Dual Luciferase Assay Kit (Promega) according to the manufacturer’s instructions. Each condition was performed in triplicate, and the luciferase activity was normalized, and an increase in luciferase activity was calculated against the experimental controls.

### 4.11. RT-PCR

A549 cells were seeded and cultured in 6-well plates until 80% confluence, followed by pre-treatment with FQs in FBS-free medium for 30 min before TGF-β and PMA treatment. Total RNA from each sample was isolated using an RNeasy Plus Mini Kit (Qiagen, Hilden, Germany) following the manufacturer’s instructions. Single-stranded cDNA was synthesized from total RNA (2 μg) by reverse transcription using a RevertAid First Strand cDNA Synthesis Kit (Fermentas, Hanover, MD, USA). Fragments specific to the examined genes were PCR-amplified in a reaction solution containing cDNA, deoxyribonucleotide triphosphates, Taq DNA Polymerase (New England Biolabs, Ipswich, MA, USA), and specific sense and anti-sense primers (5 nM). The primer sequences used for MMP-9, MMP-2, PAI-1, vimentin, and β-actin are listed in Table 1. The PCR products were resolved by 1% agarose gel electrophoresis and visualized by ethidium bromide staining. Images were captured using a Bio-Rad ChemiDoc imaging system (Bio-Rad Laboratories).

### 4.12. Statistical Analysis

All statistical analyses were performed using Prism 5 (GraphPad Software, RRID: SCR_002798, San Diego, CA, USA). The results are expressed as the mean ± SD of three independent experiments. Statistical significance was assessed using an unpaired Student’s *t*-test or analysis of variance for multiple comparison tests.

## Figures and Tables

**Table 1:** Primer sequences used in the present study.

Gene Name	Primer Sequences
MMP-9	Forward 5′-CGT GCT GAC ATC TAT GCA AT-3′ Reverse 5′-AGC TGC TCC ATT GGC ATA C-3′
MMP-2	Forward 5′-TGC ACA TCG TCC TGT GGA C-3′ Reverse 5′-GTC TCA AAC TGC TCT GAA GTG TTC-3′
PAI-1	Forward 5′-GGC TGA CTT CAC GAG TCT TTC A-3′ Reverse 5′-TTC ACT TTC TGC AGC GCC T-3′
Vimentin	Forward 5′-GAC GCC ATC AAC ACC GAG TT-3′ Reverse 5′-CTT TGT CGT TGG TTA GCT GGT-3′
β-actin	Forward 5′-CTA CAA TGA GCT GCG TGT GG-3′ Reverse 5′-AAG GAA GGC TGG AAG AGT GC-3′

MMP, matrix metalloproteinase; PAI-1, plasminogen activator inhibitor-1.

## Data Availability

Not applicable.

## Supplementary Materials

The following are available online at https://www.mdpi.com/article/10.3390/ijms222111602/s1.
