# Supplementary material for: Fluoroquinolones Suppress TGF-β and PMA-Induced MMP-9 Production in Cancer Cells: Implications in Repurposing Quinolone Antibiotics for Cancer Treatment
Source: Int J Mol Sci. 2021 Oct 27;22(21):11602. doi: 10.3390/ijms222111602 (PMC8584204; doi:10.3390/ijms222111602)
Supplement: Supplementary file 1 [file ijms-22-11602-s001.zip › ijms-1413252-supplementary.pdf]

Supplementary Materials

Fluoroquinolones Suppress TGF-β and PMA-Induced MMP-9 Production in Cancer Cells: Implications in Repurposing Quinolone Antibiotics for Cancer Treatment

Cheng-Yi Huang[2†], Jenq-Lin Yang[3†], Jih-Jung Chen[4, 5†], Shun-Ban Tai[1, 6], Yu-Hsuan Yeh[1], Pei-Feng Liu[7], Ming-Wei Lin[8], Chih-Ling Chung[1] and Chun-Lin Chen[1,9,10*]

[1] Department of Biological Sciences, National Sun Yat-Sen University, Kaohsiung 80424, Taiwan.
[2] Department of Pathology, Kaohsiung Armed Forces General Hospital, Kaohsiung 80284, Taiwan.
[3] Institute for Translational Research in Biomedicine, Kaohsiung Chang Gung Memorial Hospital, Kaohsiung 83301, Taiwan.
[4] Department of Pharmacy, School of Pharmaceutical Sciences, National Yang Ming Chiao Tung University, Linong Street, Taipei 11221, Taiwan.
[5] Department of Medical Research, China Medical University Hospital, China Medical University, Taichung 40402, Taiwan
[6] Division of Rheumatology, Immunology and Allergy, Department of Internal Medicine, Zuoying Branch of Kaohsiung Armed Forces General Hospital, Kaohsiung 81342, Taiwan.
[7] Department of Biomedical Science and Environmental Biology, Kaohsiung Medical University, Kaohsiung 80756, Taiwan.
[8] Department of Chinese Medicine, E-Da Hospital, Kaohsiung, 82445, Taiwan.
[9] Department of Biotechnology, Kaohsiung Medical University, Kaohsiung 80708, Taiwan.
[10] Graduate Institute of Natural Products College of Pharmacy Kaohsiung Medical University, Kaohsiung 80756, Taiwan.
* Corresponding author: Chun-Lin Chen, Department of Biological Sciences, National Sun Yat-sen University, Kaohsiung 80424, Taiwan. Tel: +886-7-5252000 ext 3629. Fax: +886-7-5253609. E-mail: chunlinchen@mail.nsysu.edu.tw

Contents

Figure S1. Cytotoxicity effects of fluoroquinolones in HepG2 cells S2
Figure S2. Cytotoxicity effects of TGF-β and PMA in A549 cells....................S4
Figure S3. Effects of fluoroquinolones in stability of TGF-β-stimulated MMP-9 mRNA in HepG2 cells.........S5
Figure S4. Effects of fluoroquinolones in TGF-β-stimulated MMP-9 and SBE promoters in A549 cells S5

Figure S1. Cytotoxicity effects of fluoroquinolones in HepG2 cells.

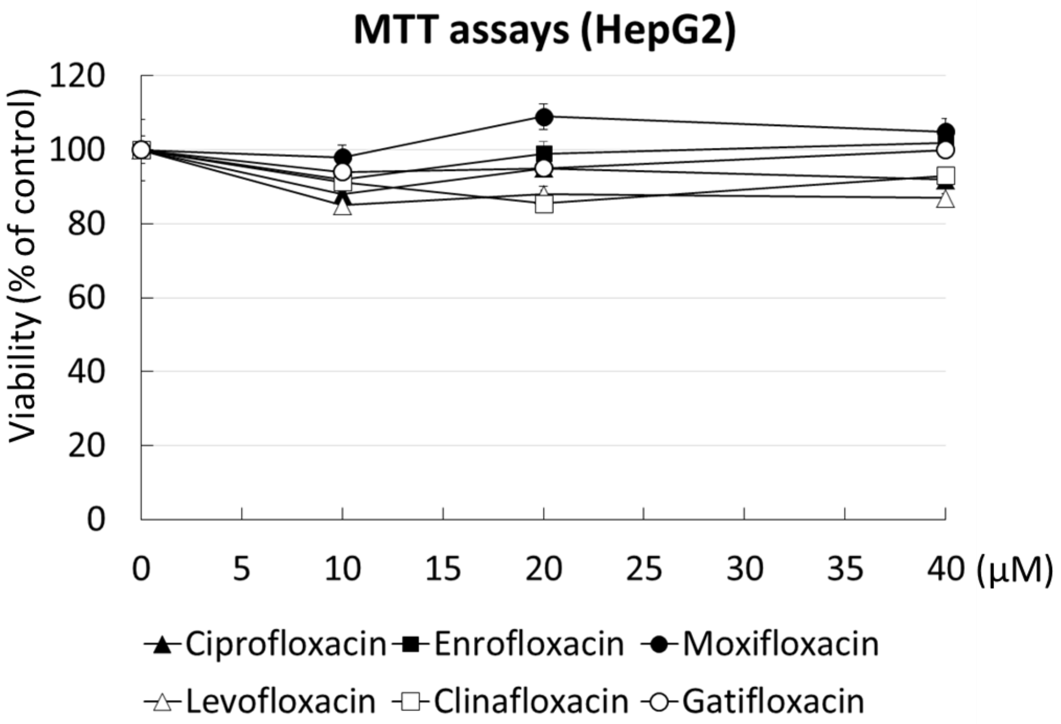

Figure S2. Cytotoxicity effects of TGF-β and PMA in A549 cells.

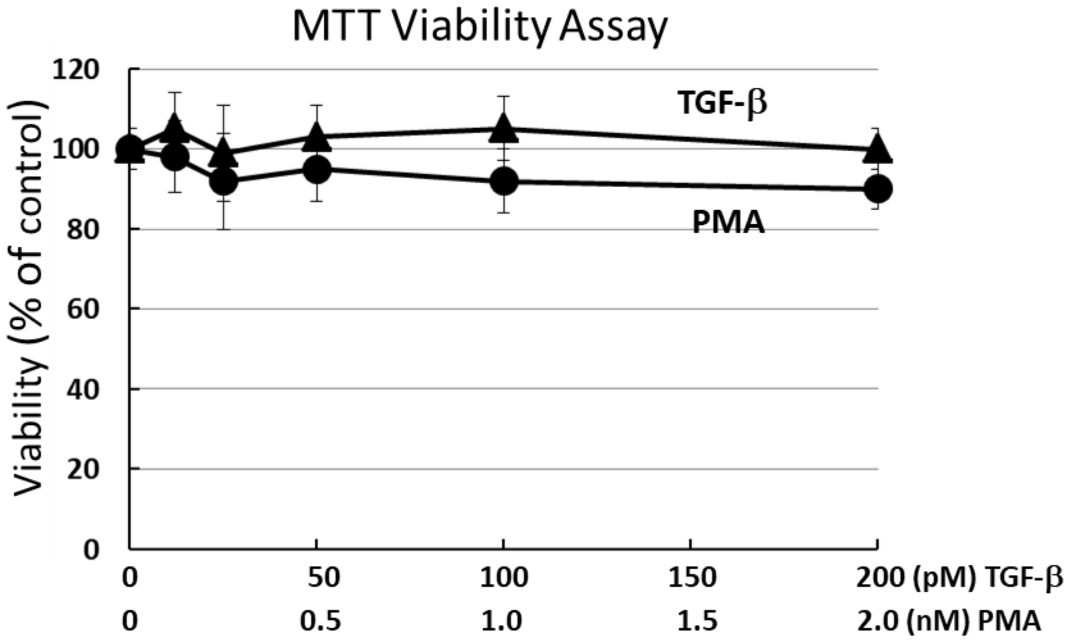

(A) HepG2 cells (1 × 10^4/well in a 96-well plate) were treated with 0–200 pM of TGF-β or 0-2nM of PMA for 48 h. Cell viability of cells was determined by the 3-(4,5-dimethylthiazol-2-yl)-2,5-diphenyltetrazolium bromide assay. Each treatment group was normalized to each un-treated control. The data (mean ± standard deviation, SD) are representative of three independent experiments; error bars indicate SD.

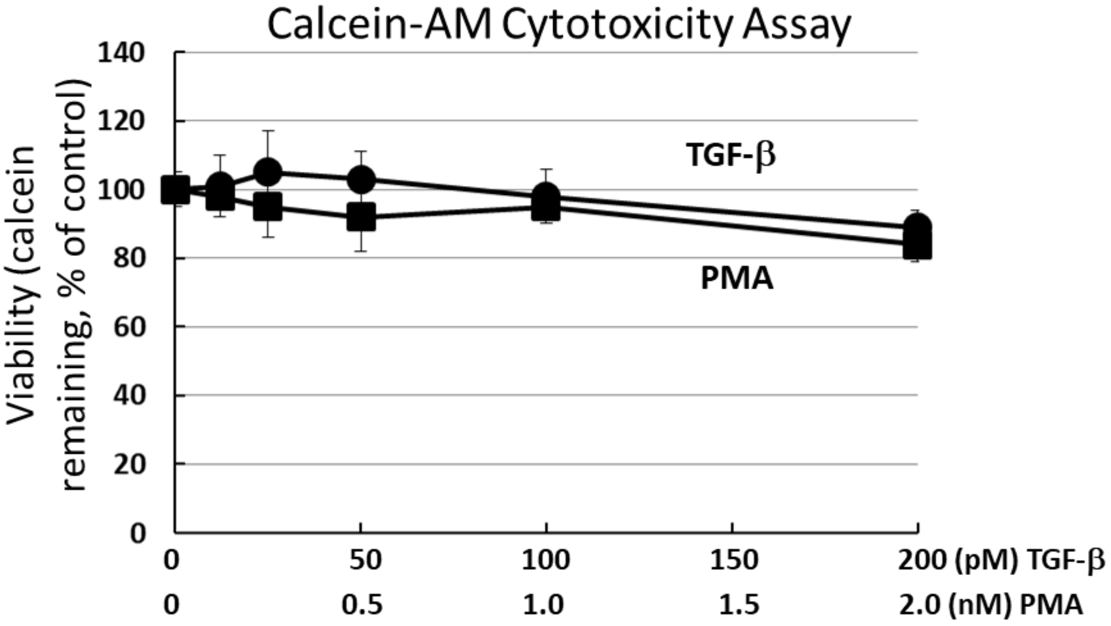

(B) Fluorometric assays on A549 cells. Dose-response relation to increasing concentrations of TGF-β or PMA on calcein-AM loaded A549. Fluorescent signal was read 45 min after TGF-β or PMA treatment. For each concentration, the fluorescence of eight wells was measured.

Figure S3. Effects of fluoroquinolones in stability of TGF-β-stimulated MMP-9 mRNA in HepG2 cells.

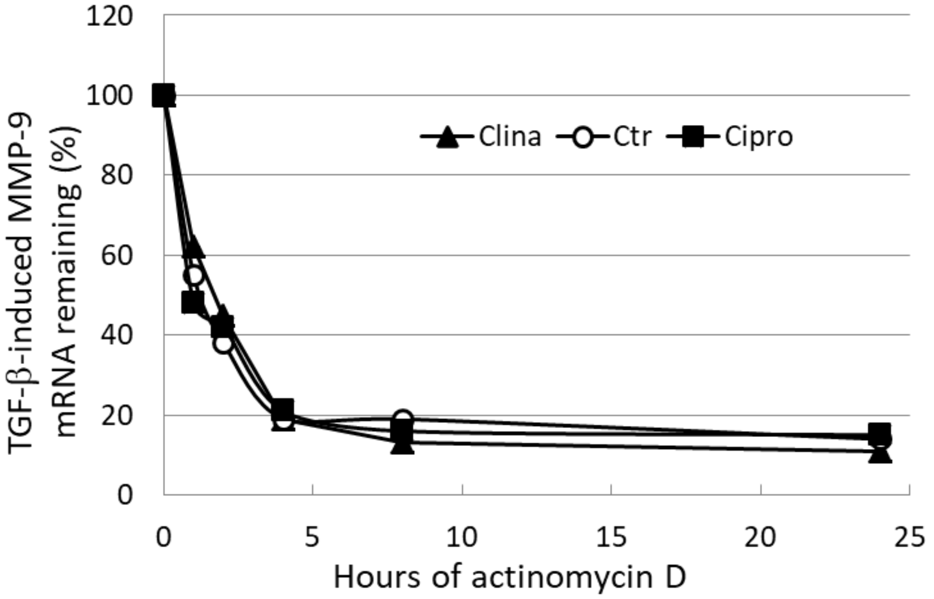

Ciprofloxacin and clinafloxacin did not change TGF-β-induced MMP-9 mRNA stability. mRNA degradation was quantified by real time RT-PCR at various times after addition of TGF-β + actinomycin D, TGF-β + actinomycin D + ciprofloxacin, or clinafloxacin. Addition of actinomycin D induced a rapid degradation of MMP-9 mRNA, which was not changed in the presence of ciprofloxacin, or clinafloxacin. Cells were stimulated with 200 pM TGF-β for 24 h, medium was replaced, and 20 μM ciprofloxacin, clinafloxacin, or DMSO as vehicle control were added for 1 h before actinomycin D chase.

Figure S4. Cytotoxicity effects of fluoroquinolones in HepG2 cells.

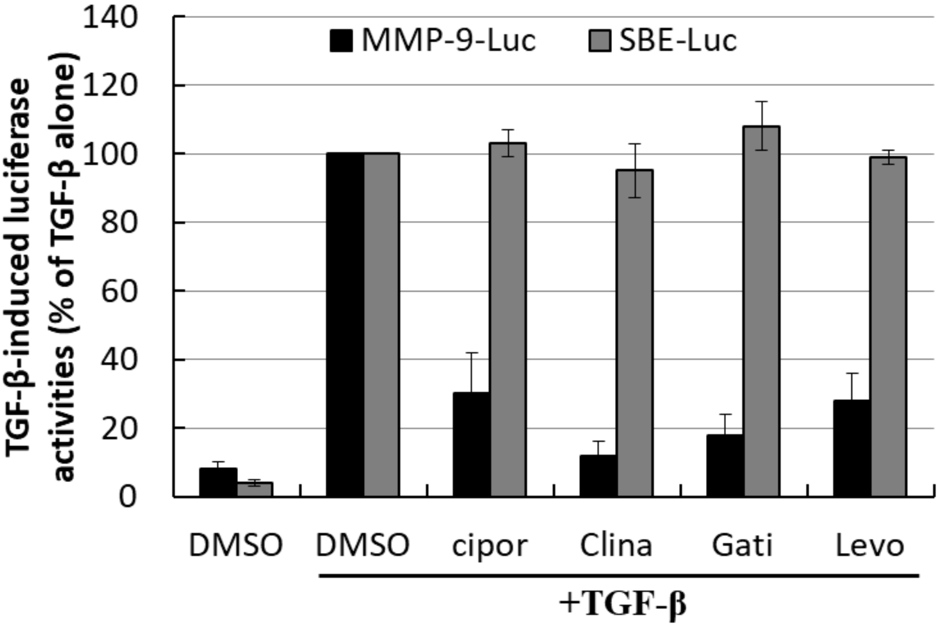

Ciprofloxacin, clinafloxacin, gatifloxacin, and levofloxacin blocked the activation of MMP-9 responsive element (MMP-9-Luc) without affecting TGF-β-responsive elements activity (SBE4-Luc).

editage

Editing Certificate

This document certifies that the manuscript listed below has been edited to ensure language and grammar accuracy and is error free in these aspects. The logical presentation of ideas and the structure of the paper were also checked during the editing process. The edit was performed by professional editors at Editage, a division of Cactus Communications. The author's core research ideas were not altered in any way during the editing process. The quality of the edit has been guaranteed, with the assumption that our suggested changes have been accepted and the text has not been further altered without the knowledge of our editors.

MANUSCRIPT TITLE

Fluoroquinolones Suppress TGF-beta and PMA-Induced MMP-9 Production in Cancer Cells: Implications in Repurposing Quinolone Antibiotics for Cancer Treatment

AUTHORS

Dr. Chun-Lin Chen

ISSUED ON

October 05, 2021

JOB CODE

QLYAN_6

THE EDITAGE Promise QUALITY GUARANTEE

Vikas Narang

Vikas Narang
Chief Operating Officer - Editage

editage

CACTUS

Editage, a brand of Cactus Communications, offers professional English language editing and publication support services to authors engaged in over 1300 areas of research. Through its community of experienced editors, which includes doctors, engineers, published scientists, and researchers with peer review experience, Editage has successfully helped authors get published in internationally reputed journals. Authors who work with Editage are guaranteed excellent language quality and timely delivery.

IMPACT SCIENCE impact.science

researcher.life

CACTUS LIFE SCIENCES lifesciences.cactusglobal.com

GLOBAL :
+1(833) 979-0061 | request@editage.com

TAIWAN :
226570306 | submitjobs@editage.com
